# p63 and p53: Collaborative Partners or Dueling Rivals?

**DOI:** 10.3389/fcell.2021.701986

**Published:** 2021-07-05

**Authors:** Dana L. Woodstock, Morgan A. Sammons, Martin Fischer

**Affiliations:** ^1^Department of Biological Sciences, The State University of New York at Albany, Albany, NY, United States; ^2^Computational Biology Group, Leibniz Institute on Aging – Fritz Lipmann Institute (FLI), Jena, Germany

**Keywords:** p53, p63, tumor suppressor, oncogene, transcription factor, pioneer factor

## Abstract

The tumor suppressor p53 and its oncogenic sibling p63 (ΔNp63) direct opposing fates in tumor development. These paralog proteins are transcription factors that elicit their tumor suppressive and oncogenic capacity through the regulation of both shared and unique target genes. Both proteins predominantly function as activators of transcription, leading to a paradigm shift away from ΔNp63 as a dominant negative to p53 activity. The discovery of p53 and p63 as pioneer transcription factors regulating chromatin structure revealed new insights into how these paralogs can both positively and negatively influence each other to direct cell fate. The previous view of a strict rivalry between the siblings needs to be revisited, as p53 and p63 can also work together toward a common goal.

## Introduction

The p53 transcription factor family comprises the three members p53, p63, and p73. Although it is evolutionarily the youngest, p53 is the eponymous member of the family. The transcription factors evolved from an ancestral *p63/p73* gene that can be found in most invertebrates (Belyi et al., 2010; Rutkowski et al., 2010). While the ancestral *p63/p73* gene protects organismal integrity and the germ line by inducing cell death upon DNA damage, higher vertebrates possess all three p53 family members with diversified functions. In particular the intricate relationship between the family members and their overlapping and opposing functions have been subject to intense research and debate.

The family matters are complicated by the fact that p53, p63, and p73 comprise multiple isoforms. The *TP53*, *TP63*, and *TP73* genes encode for major isoform groups that are controlled by distinct promoters leading to transcripts that differ in their N-terminus (Murray-Zmijewski et al., 2006). In the case of *TP63*, these isoforms are highly cell type-dependent. The long isoform TAp63 is mainly expressed in germ cells and the shorter ΔNp63 isoform is predominantly expressed in basal and stratifying epithelia. In contrast, full-length p53 is expressed across essentially all tissues. In addition to different N-termini generated through alternative promoter usage, alternative splicing leads to additional isoforms for each p53 family member that differ in their C-termini, such as α, β, and γ isoforms (Murray-Zmijewski et al., 2006).

The full-length isoforms p53α, TAp63α, and TAp73α function as haplo-insufficient tumor suppressors (Venkatachalam et al., 1998; Flores et al., 2005). In addition, p73 functions in neuronal development, multi-ciliated cell differentiation, and metabolism (Nemajerova et al., 2018). In contrast, ΔNp63 governs epidermis development (Mills et al., 1999; Yang et al., 1999) and is an oncogenic driver that is overexpressed or amplified in squamous cell carcinoma (Campbell et al., 2018; Gatti et al., 2019). The opposing directions in tumor development driven by the tumor suppressor p53α (p53 hereafter) and the proto-oncoprotein ΔNp63 involve the potential for a serious sibling rivalry. During the past two decades, the relationship between p53 and p63 has been the basis for several hypotheses and debates. Here, we provide an updated view on this relationship with an emphasis on recent genome-wide studies and we discuss whether these siblings might get along as much as they fight.

## History Fueled a Sibling Rivalry

**FIGURE 1:**
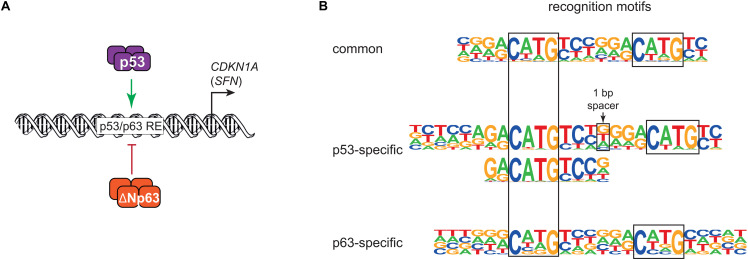
**(A)** Simplified model of transcriptional repression by ΔNp63 and its dominant negative effect on p53, largely based on Westfall et al. (2003). **(B)** DNA motifs recognized either by both p53 and ΔNp63 (common) or solely by p53 or ΔNp63. Major motifs taken from Riege et al. (2020).

In 1998, the discovery of p63 laid the foundation for a history of sibling rivalry with its more famous sibling p53. The first study of p63 found that both its full-length isoform TAp63 and the shorter ΔNp63 can bind to DNA motifs that are similar to those of p53, but only TAp63 harbored a transactivation domain (TAD). In contrast to TAp63, ΔNp63 lacked a canonical TAD and was found to confer negative effects over other p53 family members and its own isoforms (Yang et al., 1998). The function of ΔNp63 as dominant negative regulator of its family members was fueled by the initial and additional reporter assays using exogenous expression of different isoforms of p53 family members (Yang et al., 1998; Westfall et al., 2003). While Yang et al. (1998) used a minimal promoter containing multiple p53 binding sites to drive a β-galactosidase reporter, Westfall et al. (2003) employed promoter regions of *CDKN1A* (p21) containing known p53 binding sites to drive a luciferase reporter. Both studies showed that high expression of ΔNp63 was associated with a reduced ability of p53 to *trans*-activate the reporter genes, highlighting a potential sibling rivalry (Figure 1A).

## DNA Recognition Sequence Specificity

The p53 family shares a highly conserved DNA binding domain (DBD) through which its members bind to very similar DNA motifs. Consequently, p53, p63, and p73 share many binding sites, but they also bind to many unique sites (Lin et al., 2009; McDade et al., 2014; Riege et al., 2020). In agreement with their ability to also bind to unique genomic sites, differences in their DBDs have been reported with regard to thermostability, hydrophobic potentials (Enthart et al., 2016), zinc-coordination (Lokshin et al., 2007), and redox sensitivity (Tichý et al., 2013). In addition to small differences in their DBD, the different C-terminal domains of the p53 family may affect their DNA binding specificity (Sauer et al., 2008; Laptenko et al., 2015). For example, p53 is well-established to bind to two decameric half sites that both harbor the sequence RRRCWWGYYY (R = A/G; W = A/T; Y = C/T). The substantial number of unique genomic sites bound by p53 and p63 motivated a series of studies that investigated potential differences in their DNA recognition motifs. Using either systematic evolution of ligands by exponential enrichment (SELEX) (Ortt and Sinha, 2006; Perez et al., 2007) or high-throughput analyses of chromatin immunoprecipitation (ChIP-seq) (Yang et al., 2006; Kouwenhoven et al., 2010; McDade et al., 2012) led to the identification of p63 recognition motifs with high similarity to p53 motifs that sill showed some uniqe characteristics. These unique characteristics, however, differed substantially between the studies. A recent study addressed the question of p53 and p63-specific DNA recognition motifs using a meta-analysis of ChIP-seq datasets combined with an iterative *de novo* motif search approach (Riege et al., 2020). The results imply that p53 relies on one CWWG core motif with flanking regions, while p63 relies on two CNNG (N = A/C/G/T) core motifs with little importance of flanking regions. These findings support and expand one of the models established earlier (McDade et al., 2014) and explain a substantial number of genomic regions that are bound by only p53 or p63 (Riege et al., 2020; Figure 1B). DNA recognition motifs alone, however, cannot explain productive binding of p53 or p63, which occurs when p53 or p63 bind to a genomic region that functions as a *cis*-regulatory region to transcriptionally regulate a proximal or distal gene that is linked to it. In fact, p53 and p63 regulate largely non-overlapping gene sets (Gallant-Behm et al., 2012; Riege et al., 2020), which indicates that additional layers of regulation play an important role in p53 and p63-mediated gene regulation.

## Engaging Chromatin – The Pioneer Role of the p53 Family

**FIGURE 2:**
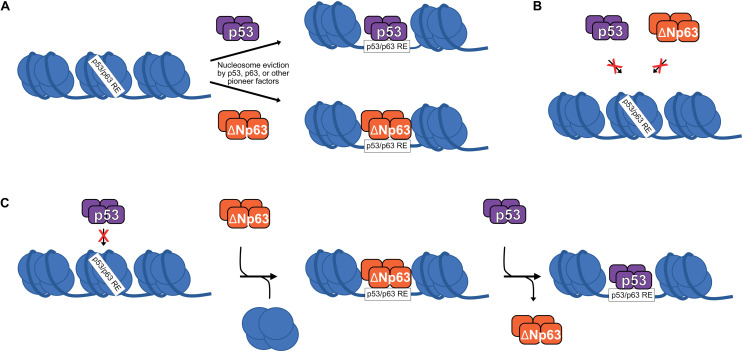
**(A)** p53 and ΔNp63 can act as pioneer factors to evict/remodel nucleosomes and facilitate DNA accessibility, although other pioneer factors are likely responsible for nucleosome eviction at the majority of p53/ΔNp63 binding sites. **(B)** Certain nucleosome contexts, such as when the p53/p63 RE motif is near the nucleosome dyad, are recalcitrant to p53 and/or ΔNp63 pioneer factor activity and remain unbound (Yu and Buck, 2019; Yu et al., 2021). **(C)** In epithelial cell types, p53 DNA binding can be facilitated by ΔNp63′s pioneer factor activity, although specific biochemical mechanisms are still being fully elucidated.

Generally, transcription factors bind to nucleosome-free DNA and are inhibited by nucleosomes. This rule is broken by pioneer transcription factors, which can bind to nucleosomal DNA either sequence-dependent or independent (Zaret and Mango, 2016). Numerous recent studies suggest that the p53 family of transcription factors are pioneer transcription factors (Sammons et al., 2015; Yu and Buck, 2019; Yu et al., 2021; Figure 2A). Indeed, differential pioneer activity of p53 and p63 has the potential to explain some of the observed gene regulatory differences between these two siblings.

p53 can bind to nucleosomal DNA, although this strongly depends on the specific location of the response element relative to the nucleosome dyad (Sahu et al., 2010; Yu and Buck, 2019). ChIP-seq studies suggest that about 50% of p53 binding sites are nucleosome-occupied, but to date, the role of nucleosome-bound p53 in transcription has only been studied at a single gene level (Espinosa and Emerson, 2001; Laptenko et al., 2011). A subset of p53 binding sites display *de novo* DNA accessibility and potential enhancer activity upon p53 binding, suggesting a requirement for p53 pioneer activity (Younger and Rinn, 2017), although p53 is not required for accessibility at the majority of its binding sites (Karsli Uzunbas et al., 2019). The full activity of p53-bound regulatory regions depends on other factors, such as SP1 and the AP-1 family (Daino et al., 2006; Catizone et al., 2020), but whether they facilitate DNA accessibility or other aspects of transcriptional activation is not fully understood.

Given the high sequence and functional homology to p53, perhaps it is unsurprising that p63 has also emerged as a pioneer transcription factor with similar nucleosomal constraints (Yu et al., 2021). Control of cell type-specific chromatin accessibility and gene expression is a feature shared by many other pioneer transcription factors (Iwafuchi-Doi and Zaret, 2014, 2016; Zaret and Mango, 2016). Unlike p53, p63 has a widespread role in establishing and maintaining accessible DNA at transcriptional regulatory regions associated with epithelial cell maturation (Bao et al., 2015; Kouwenhoven et al., 2015; Qu et al., 2018; Li et al., 2019; Lin-Shiao et al., 2019). The identification of p63 as a pioneer factor for epithelial-specific regulatory elements provides a direct molecular connection to the long-known genetic requirement for p63 in epithelial biology. We are just beginning to understand the specific contexts in which p53 and p63 use their pioneer factor activity, or don’t (Figures 2A,B). Nucleosomes, and chromatin structure in general, remain a potent regulator of transcription factor activity, including the pioneers p53 and p63.

## p53 and p63 – Dueling Rivals?

The initial model of ΔNp63 functioning as repressor and dominant negative regulator of its siblings was based on its missing TAD (Yang et al., 1998), and was fueled by experiments using reporter gene assays and exogenous expression of p63 or its siblings (Yang et al., 1998; Westfall et al., 2003; Figure 1A). Wild-type p53 and p63 are unlikely to form oligomers *in vivo* (Davison et al., 1999; Gaiddon et al., 2001), ruling out the formation of p53:p63 heterotetramers as a mechanism for any dominant negative activity. Notably, ΔNp63 was found to harbor alternative TADs that enable it to *trans*-activate genes (King et al., 2003; Helton et al., 2006). Results from the first integration of ΔNp63 ChIP-seq and transcriptomic data revealed ΔNp63 genome binding to be associated largely with *trans*-activation (Yang et al., 2006). Transcriptome analyses revealed that p63 and p53-regulated genes show only very little overlap (Gallant-Behm et al., 2012), which was inconsistent with ΔNp63 functioning as a negative regulator of its siblings. A broad meta-analysis of ChIP-seq and transcriptomic data corroborated that p63 and p53 regulate largely non-overlapping gene sets, and argue that ΔNp63 is more likely to activate than to repress its target genes (Riege et al., 2020). While genome-wide data revealed an essentially exclusive *trans*-activator function for p53 (Allen et al., 2014; Fischer et al., 2014, 2016a; Sullivan et al., 2018; Sammons et al., 2020), a *trans*-repressor function could not be ruled out for ΔNp63 (Yang et al., 2006; Riege et al., 2020). Interestingly, during epithelial cell maturation, ΔNp63 represses early surface ectoderm gene promoters presumably through chromatin looping-mediated recruitment of repressive histone modifications (Pattison et al., 2018), providing a novel mechanism for p63-mediated repression. The specific impact of this, and the ability of ΔNp63 to recruit negative transcriptional regulators like histone deacetylases and histone variants (LeBoeuf et al., 2010; Ramsey et al., 2011; Gallant-Behm et al., 2012), on p53 activity remains to be fully explored.

The predominant function of ΔNp63 as *trans*-activator and the small overlap between p63 and p53-regulated genes suggest any model of ΔNp63 functioning strictly as a dominant negative regulator of p53 cannot be upheld. p53 and p63 have many opportunities to regulate each other, given the high overlap in genomic binding locations (McDade et al., 2014; Riege et al., 2020). Importantly though, the 180 high-confidence direct ΔNp63 targets and 343 high-confidence direct p53 targets identified by meta-analyses of ChIP-seq and transcriptomic data (Fischer, 2017; Riege et al., 2020) contain an overlap of only 23 genes, 19 (>82%) of which are commonly up-regulated by p53 and ΔNp63.

Having this much organized data at hand, what do we know we about *CDKN1A* and *SFN*, the genes that initially fueled p53 and p63′s history of sibling rivalry (Westfall et al., 2003)? *CDKN1A* has been identified in essentially all p53 ChIP-seq and transcriptome profiling datasets as a direct p53 target gene. While ΔNp63 can bind to the *CDKN1A* promoter, only 4 and 3 out of 16 datasets on ΔNp63-dependent gene regulation identified ΔNp63 to significantly up and down-regulate *CDKN1A*, respectively. *SFN* was identified as a direct p53 and ΔNp63 target that is typically up-regulated by both siblings (Fischer, 2017; Riege et al., 2020). Together, these data suggest that the view of ΔNp63 functioning as a potent negative regulator of p53 can be rejected and replaced with a context-dependent model where ΔNp63 functions as either a *trans*-activator or a repressor depending on cell type and binding location. Future work will undoubtedly be focused on better defining the context for these opposing activities.

## p53 and p63 – Collaborative Partners?

Although they appear to regulate a mostly unique set of target genes and have non-overlapping cellular roles, genetic evidence suggests that p53 and p63 cooperate to regulate DNA damage-induced apoptosis in mouse embryonic fibroblasts (Flores et al., 2002). p53 binds to cell cycle arrest target genes like *CDKN1A* in the absence of p63, but was unable to interact with promoters of the pro-apoptotic genes *NOXA* and *BAX* (Flores et al., 2002). The specific molecular mechanisms regulating this apparent collaborative effort for p53 and p63 are still unknown, but the recent identification of ΔNp63 as a pioneer transcription factor provides one possibility. p53 genomic binding and gene regulatory activity is expanded in epithelial cell types (McDade et al., 2014; Sammons et al., 2015; Nguyen et al., 2018; Karsli Uzunbas et al., 2019). These novel p53 binding sites have epithelial cell-specific DNA accessibility, have chromatin modifications associated with active enhancers, and, importantly, are strongly bound by ΔNp63. Inhibition of ΔNp63 leads to depletion of active transcription-associated hallmarks at these sites and diminishes the ability of p53 to activate nearby genes (Karsli Uzunbas et al., 2019). These sites are nucleosome rich with little to no DNA accessibility in the absence of ΔNp63 (Thurman et al., 2012), and p53 does not bind these sites in non-epithelial cell types (Nguyen et al., 2018; Karsli Uzunbas et al., 2019). Presumably, this is due to the ability of ΔNp63 to mediate local chromatin accessibility with its pioneer factor activity (Sammons et al., 2015; Karsli Uzunbas et al., 2019). Modulation of local and distal chromatin states, be it to facilitate transcriptional activation (Fessing et al., 2011; Bao et al., 2015; Li et al., 2019; Catizone et al., 2020) or repression (Gallant-Behm et al., 2012; Pattison et al., 2018) appears to be a key function of ΔNp63 and paves the way for the field to resolve many of the incongruent observations regarding ΔNp63′s influence on p53.

## Opposing Directions in Tumor Development

While current data suggest that p53 and ΔNp63 are more likely to cooperate than to compete at DNA, they remain functionally quite different. Perhaps most importantly, ΔNp63 promotes while p53 restricts cellular growth. As a consequence, ΔNp63 is a key oncogenic driver in squamous cell carcinoma (Campbell et al., 2018; Gatti et al., 2019) while p53 is the best-known tumor suppressor. The context-dependent tumor suppressor role of p63 (Flores et al., 2005; Keyes et al., 2006) appears to be largely reflected by the tumor suppressive function of the TAp63 isoform that induces apoptosis and senescence (Gressner et al., 2005; Suh et al., 2006; Guo et al., 2009). The contrary direction driven by p53 and ΔNp63 in tumor development can be explained on the one hand by their unique target genes. While unique direct ΔNp63 target genes encode for several proteins that promote squamous cell cancer growth, inflammation, and invasion (Somerville et al., 2018, 2020; Riege et al., 2020), unique p53 target genes encode inducers of cell cycle arrest and apoptosis (Fischer, 2017). On the other hand, there is the large set of cell cycle genes differentially regulated by p53 and ΔNp63 (Riege et al., 2020). p53 employs its direct target gene *CDKN1A*, encoding the cyclin-dependent kinase inhibitor p21, to reactivate the cell cycle *trans*-repressor complexes DREAM and RB:E2F (Fischer et al.,2016a,b; Schade et al., 2019; Uxa et al., 2019). While it is not completely understood how ΔNp63 up-regulates cell cycle genes, it was suggested to inhibit the p21–p130 (DREAM) axis (Truong et al., 2006; McDade et al., 2011) and to *trans*-activate multiple cell cycle genes directly (Riege et al., 2020). We have a detailed picture of how p53 down-regulates cell cycle genes and sustains cell cycle arrest (Schade et al., 2019; Uxa et al., 2019). It remains unresolved, however, whether the regulation of cell cycle genes is cause or consequence of the growth-promoting function of ΔNp63, as it is well established that high expression of cell cycle genes is associated with cancer and worse prognosis (Whitfield et al., 2006). Together, the unique direct p53 and ΔNp63 target genes as well as the differential regulation of cell cycle genes elicited by p53 and ΔNp63 offer a partial, but direct, explanation for their opposing functions in tumor development.

## Discussion

Sibling rivalry can happen in any family and it is no different for the p53 transcription factor family. p63 was within p53′s considerably large shadow from the beginning, but p63 has started to step into the light with the discoveries of its clear genetic requirement during development, regulation of a pro-epithelial gene network, and pioneer activity. Now, what are the key questions that need to be addressed regarding the collaboration and competition between p53 and p63?

The other factors and precise context required for p53 and ΔNp63 to elicit productive binding to DNA and to regulate distinct target genes remain unclear. Although ΔNp63 occupies most sites that can be bound by p53, it appears to affect only a very small subset of the associated genes. It is unknown how ΔNp63 distinguishes between the many sites it activates, the smaller number of sites it represses, and the majority of sites it appears to not affect transcriptionally. Along those lines, when and where are p53 and ΔNp63 pioneer factors? The context and the extent to which pioneer activity is required for p53 family function remains an important and active area of investigation. And how collaborative is their oft-forgotten sibling p73?

Despite beginning their relationship as rivals, p53 and ΔNp63 appear to cooperate with each other when mutually beneficial. Identifying the situations when these two transcription factors are collaborators and when they are competitors may provide a blueprint to better understand mechanisms of how transcription factor families that share binding sites and target genes coordinate their efforts.

## Author Contributions

MS and MF conceptualized the manuscript and prepared the figures. DW, MS, and MF performed the literature review, provided an outline, wrote and edited the manuscript, and approved the submitted version. All authors contributed to the article and approved the submitted version.

## Conflict of Interest

The authors declare that the research was conducted in the absence of any commercial or financial relationships that could be construed as a potential conflict of interest.
